# Parenting styles and smartphone dependence among college students: the serial mediating roles of self-control and gratitude

**DOI:** 10.3389/fpsyg.2026.1874356

**Published:** 2026-09-23

**Authors:** Xin Li, Kien Tsong Chau, Wan Ahmad Jaafar Wan Yahaya, Xiaohui Sun

**Affiliations:** 1 Department of Computer Science, Linfen Vocational and Technical College, Linfen, China; 2 Centre for Instructional Technology and Multimedia, Universiti Sains Malaysia, Penang, Malaysia; 3 Office of Academic Affairs, Xi’an Fanyi University, Xi’an, China

**Keywords:** college students, gratitude, parenting styles, self-control, serial mediation, smartphone dependence

## Abstract

**Background:**

Smartphone dependence among college students has become an increasingly important concern because of its associations with physical and mental health and academic functioning. Parenting styles, as a core component of the family microsystem, may exert enduring influences on psychological and behavioral development. However, the psychological mechanisms linking parenting styles to smartphone dependence remain insufficiently understood.

**Objective:**

This study aimed to investigate the relationship between parenting styles and smartphone dependence among college students, and to examine the serial mediating roles of self-control and gratitude.

**Methods:**

A cross-sectional survey design was adopted, involving 776 Chinese college students. Measurements were conducted using the Parenting Styles Questionnaire, Smartphone Addiction Index, Self-Control Scale, and Gratitude Scale. Data were analyzed using SPSS 22.0 and the PROCESS macro for correlation analysis, regression analysis, and serial mediation effect testing.

**Results:**

Parental rejection and overprotection significantly positively predicted smartphone dependence, while the direct effect of emotional warmth was not significant. Both self-control and gratitude played significant mediating roles between parenting styles and smartphone dependence. A significant serial mediating effect was observed along the path “parenting styles influence self-control, which shapes gratitude and subsequently affects smartphone dependence,” with effect sizes ranging between 2.3 and 2.4%.

**Conclusion:**

Parenting styles were associated with college students’ smartphone dependence through a serial pathway involving self-control and gratitude. Positive parenting dimensions were associated with stronger self-control and gratitude, whereas negative parenting dimensions were associated with weaker psychological resources and greater smartphone dependence. These findings provide a theoretical basis for future research and may inform family- and self-regulation-oriented interventions.

## 1 Introduction

Smartphones are central to adolescents’ and young adults’ communication, learning, access to information, and entertainment. While high levels of use are not necessarily problematic, problematic use of smartphones is better defined as loss of control and interference with functioning in study, sleep, relationships or wellbeing (Bianchi and Phillips, 2005; Walsh et al., 2011). The distinction is particularly relevant in China given the scale of mobile access. By June 2024, China had nearly 1.10 billion Internet users, with mobile phones being the dominant mode of Internet access (CNNIC, 2021).

In this highly interconnected context, college students deserve special attention because emerging adulthood is marked by greater autonomy, academic demands, changes in peer networks, identity exploration, and less direct parental monitoring. Smartphones can be used to aid coursework, social connection, and coping, but their constant accessibility can also lead to repeated checking and immediate digital rewards in response to stress, boredom, or interpersonal difficulties (Guo, J., et al., 2025; Song et al., 2025). The key distinction, therefore, is not between smartphone access and dependence, but between normal, goal-directed use and persistent, poorly regulated use that leads to functional impairment. Prior research has shown that the extent of problematic smartphone use among university students varies widely, and this is partly due to differences in measurement, study settings, and cut-off points (Zhao et al., 2021). Studies have suggested that problematic smartphone use is associated with negative emotion, fear of missing out (FOMO), low perceived social support and poor self-regulation in Chinese college students (Guo, J., et al., 2025; Song et al., 2025; Zhong et al., 2025). In addition, excessive use has been linked to sleep disruption, musculoskeletal and visual strain, anxiety, depression, loneliness, a reduced sense of belonging, decreased happiness, and poorer academic performance (Akinci and Durmuş, 2025; Elamin et al., 2024; Peng et al., 2025; Zhong et al., 2025). Hence, this dependence on smartphones should be distinguished from regular but controlled usage. While the terminology used to describe these features differs between studies, impaired control and functional impairment are core features of addiction-like smartphone use (Bianchi and Phillips, 2005; Walsh et al., 2011). In this study, the conceptualisation of smartphone dependence was persistent compulsive engagement in smartphone-mediated activities that are difficult to control and have negative academic, social, or psychological consequences. This operational definition is based on addiction-like symptoms assessed with the Mobile Phone Addiction Index and does not denote a clinical diagnosis.

### 1.1 Parenting styles and smartphone dependence among college students

While college students have more independence and less explicit parental supervision, family socialisation may still influence behavior through internalized expectations, attachment security, habitual emotion-regulation strategies, and self-regulatory capacities (Li et al., 2019; Xin et al., 2022). Parenting style refers to relatively stable patterns of parent–child interactions perceived in terms of warmth, rejection, control, and behavioral guidance. Such experiences may shape the way emerging adults evaluate stress, seek support, delay gratification, and regulate impulses. However, parenting does not operate in isolation: current peer relationships, academic stress, social support, and the digital environment may reinforce, buffer, or modify earlier family influences (Guo, J., et al., 2025; Zhong et al., 2025).

There is evidence that parenting styles are associated with smartphone dependence, although the strength and direction of these associations vary across dimensions (He et al., 2024; Lian et al., 2016; Xin et al., 2022). Rejection and hostility may contribute to chronic emotional insecurity and negative self-evaluations. In college, such vulnerabilities may increase reliance on online reassurance or distraction when students encounter academic or interpersonal stress. In contrast, emotional warmth and appropriate autonomy support may foster secure relationships and adaptive self-regulation, thereby reducing reliance on smartphones as a coping resource (Li et al., 2022). These developmental pathways are probabilistic rather than deterministic, and their expression may depend on the social resources and demands students encounter in the university environment.

Parental overprotection refers to intrusive control and excessive decision making on behalf of the child. Such patterns may limit opportunities to develop autonomy and coping skills, potentially leaving students less confident in managing problems independently (Lian et al., 2016). When faced with academic pressure or interpersonal conflict, these students may be more likely to turn to readily accessible smartphone activities for distraction or avoidance. However, supportive peers, mentoring, and campus resources may provide alternative sources of coping, illustrating that the influence of family experiences may be shaped by the current university environment.

From a theoretical perspective, the association between parenting styles and smartphone dependence can be interpreted through Problem Behavior Theory (PBT), which locates problem behavior within the interplay of the perceived social environment, personality-related characteristics, and behavior (Richard Jessor, 1991). In the present context, perceived parenting represents one component of the social environment, whereas self-control and gratitude are examined as psychological characteristics that may be associated with behavioral choices. This framework supports testing whether parenting dimensions, self-control, gratitude, and smartphone dependence are statistically related, while recognizing that the present cross-sectional design cannot establish a developmental sequence.

### 1.2 The mediating role of self-control

Self-control refers to the capacity to inhibit impulses, delay gratification, and regulate behavior in accordance with longer-term goals. Low self-control has been consistently associated with problematic smartphone use and may represent an important pathway through which social and interpersonal conditions are related to smartphone dependence (Guo, S., et al., 2025; Servidio, 2019; Zhong et al., 2025). Because smartphones provide readily accessible and continuously available digital rewards, students with lower self-control may find it more difficult to regulate checking, gaming, or short-video use when these activities conflict with academic or sleep-related goals.

Accordingly, self-control may serve as a protective factor against smartphone dependence. Students with higher self-control are better able to regulate smartphone use, resist immediate rewards, and prioritise longer-term goals (Khang et al., 2013). In contrast, students with lower self-control may be more vulnerable to repeated and impulsive smartphone use, which can gradually develop into impaired control. Consistent with this view, previous studies have reported a negative association between self-control and smartphone dependence among college students, with lower self-control being associated with higher levels of problematic use (Li et al., 2019; Servidio, 2019).

Parenting styles may also contribute to the development of self-control by shaping opportunities for autonomy, rule internalization, and emotion regulation. Warm and appropriately structured parenting can provide children with opportunities to practice behavioral regulation, whereas excessive control or overprotection may restrict opportunities for independent self-management. A three-level meta-analysis identified systematic associations between parenting and self-control in adolescence (Li et al., 2019), while longitudinal and mediation studies suggest that self-control may transmit the influence of family and peer contexts on problematic smartphone use (Finkenauer et al., 2005; Guo, S., et al., 2025). Taken together, these findings provide support for self-control as a potential mediator linking parenting styles with smartphone dependence.

### 1.3 The mediating role of gratitude

In addition to self-control, gratitude may represent another psychological pathway linking parenting styles with smartphone dependence. Gratitude refers to the recognition and appreciation of benefits received from others and the willingness to respond positively to such experiences (Dickens and DeSteno, 2016). Previous research suggests that gratitude is associated with both parenting experiences and problematic technology-related behaviors, indicating its potential protective role.

Parenting styles may contribute to the development of gratitude by shaping children’s experiences of warmth, support, and interpersonal reciprocity. Positive parenting provides repeated opportunities for children to experience and recognise care, which may facilitate the internalization of grateful attitudes through emotional experience, behavioral modelling, and social learning (Barnett et al., 2021). In contrast, rejection and excessive control may weaken children’s perceptions of interpersonal support and reduce opportunities to recognise and appreciate positive experiences (Saleem et al., 2021). These findings suggest that parenting styles may be associated with individual differences in gratitude that persist into emerging adulthood.

Gratitude may, in turn, reduce vulnerability to smartphone dependence by promoting positive psychological resources and adaptive engagement with the real-world environment. According to Fredrickson’s (2001a, b) Broaden-and-Build Theory of Positive Emotions, positive emotional experiences can broaden individuals’ cognitive and behavioral repertoires and facilitate the development of psychological resources. In this context, gratitude may reduce reliance on smartphone-based rewards by directing attention toward positive experiences in everyday life, supporting emotional regulation, and encouraging interpersonal engagement. Consistent with this perspective, gratitude has been associated with lower levels of problematic online behaviors, including online gaming addiction (Gao et al., 2023), while smartphone addiction has also been associated with lower levels of gratitude and interpersonal virtues (Lian et al., 2016).

Overall, the existing literature suggests that parenting styles may be indirectly associated with smartphone dependence through gratitude. Positive parenting may foster gratitude and related psychological resources, whereas adverse parenting experiences may be associated with lower gratitude and greater vulnerability to problematic smartphone use. Therefore, gratitude may serve as a potential mediator in the association between parenting styles and smartphone dependence.

### 1.4 The serial mediating roles of self-control and gratitude

Self-control and gratitude may also operate sequentially rather than independently. Existing evidence suggests that self-control and gratitude are positively related, while each has also been associated with problematic smartphone or online behavior (DeSteno et al., 2014; Gao et al., 2023; Servidio, 2019). Greater self-control may help individuals regulate immediate impulses and attend to longer-term interpersonal benefits, whereas gratitude may support engagement with valued relationships and experiences. These associations provide a theoretical rationale for testing a serial statistical model in which perceived parenting is associated with self-control, self-control is associated with gratitude, and gratitude is associated with smartphone dependence. The proposed ordering is a hypothesis to be tested longitudinally rather than a causal sequence established by the present data.

Taken together, the literature provides a basis for testing a serial mediation model in which parenting styles are associated with smartphone dependence through the sequential pathways of self-control and gratitude.

### 1.5 The present study

The proposed model combines ecological systems theory, Problem Behavior Theory, self-regulation perspectives and the broaden-and-build account of positive emotion. Parenting styles represent a family-microsystem influence. Self-control is a regulatory capacity relevant to responses to immediate digital rewards. Gratitude is a positive cognitive-affective resource that directs attention toward valued relationships and experiences. Smartphone dependence is the behavioral outcome. Problem Behavior Theory links environmental and personality systems to behavior, and self-regulation and positive-emotion perspectives provide the rationale for the proposed ordering of self-control and gratitude in the serial pathway. Academic and peer contexts are examined as contextual influences that may amplify or buffer these associations rather than as alternatives to family influence. Therefore, the current study examined the mediating effect of self-control and gratitude in the association between parenting styles and smartphone dependence, respectively and sequentially (Figure 1).

**Figure 1:**
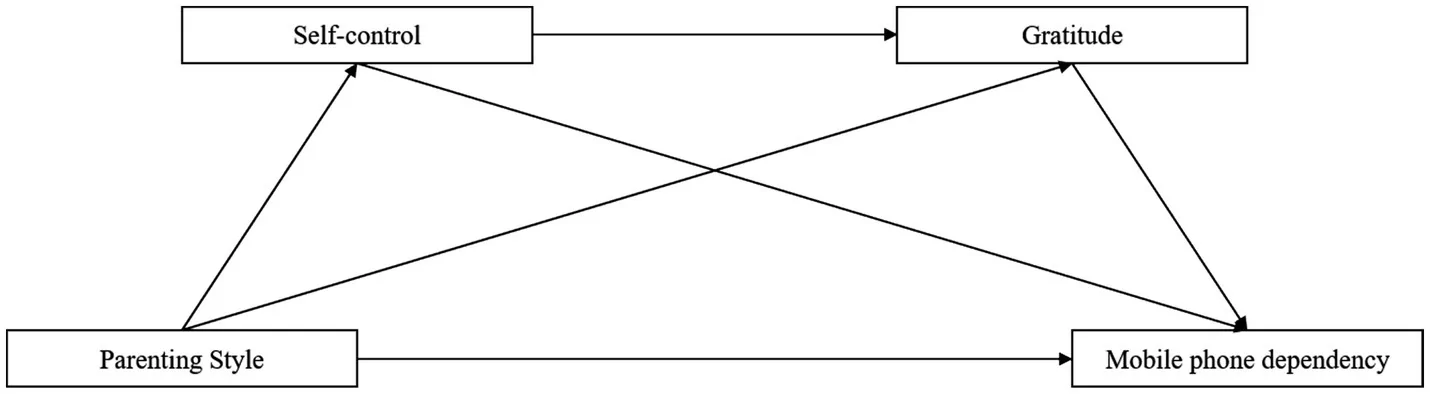
Theoretical model.

## 2 Materials and method

### 2.1 Research hypotheses and study framework

The current study investigated the mediating roles of self-control and gratitude in the association between parenting styles and smartphone dependence, including the possibility of serial mediation. Three hypotheses were tested based on the previous theory and empirical literature. (1) Parenting styles would be significantly related to smartphone dependence, self-control and gratitude. (2) Self-control would be significantly related to gratitude and smartphone dependence, and gratitude would be significantly related to smartphone dependence. (3) Self-control and gratitude would serially mediate the relationships between parenting styles and smartphone dependence.

This study examined direct and indirect associations between smartphone dependence and three dimensions of parenting, namely parental rejection, emotional warmth and overprotection thru self-control and gratitude. It addressed three gaps in the literature: insufficient integration of family, self-regulatory, and positive-psychology explanations, insufficient examination of self-control and gratitude in a single sequential model, and insufficient evidence on these pathways among Chinese college students. Thus, the model assesses the extent to which perceived family experiences might be linked to a proximal digital behavior thru psychological resources that may be modifiable (Figure 1).

### 2.2 Participants and procedure

The present study surveyed undergraduate students from first year to fourth year at three universities in mainland China. Questionnaire data were collected between January and April 2022 by using a grade-stratified convenience sampling method to recruit participants. Objective constraints prevented us from conducting a nationwide probability-based stratified sampling due to the lack of a comprehensive sampling frame of all Chinese college students, and practical limitations in terms of research funding, personnel, and cross-institutional collaboration. Therefore, a grade-stratified convenience sampling technique was implemented, with the recruitment process balancing the number of participants across grade levels, to ensure adequate sample sizes for students in each academic year. This approach helped to reduce, at least partially, the possible sampling bias resulting from uneven grade distribution. However, it must be noted that grade-stratified convenience sampling is a non-probability sampling method. Although this approach improves the sample representation of the grade dimension, the self-selection bias of voluntary participation cannot be eliminated, which is a limitation of convenience sampling in general. All subsequent regression analyses and mediation models controlled for observable demographic confounders (gender, age, hukou [household registration] type, and only-child status) by including them as covariates in the model. However, covariate adjustment can only account for observed demographic differences and cannot correct for unobserved self-selection biases due to non-random recruitment.

All participants volunteered and questionnaires were completed by eligible undergraduate students from participating institutions. Total 814 questionnaires were distributed. After data screening, invalid questionnaires were excluded (*n* = 38), resulting in 776 valid questionnaires and an effective response rate of 95.3%. The participants’ ages ranged from 18 to 25 years (*M* = 20.34, SD = 1.21). All participants were currently enrolled undergraduates; the upper age limit of 25 years is within the reasonable range of emerging adulthood as age variation within this population may legitimately be attributed to delayed enrolment, academic interruption, or extended study periods. There were 247 males (31.8%) and 528 females (68.0%), 284 only-child participants (36.6%) and 491 participants with siblings (63.3%), 403 participants with urban hukou (51.9%) and 372 participants with rural hukou (47.9%). The sample had a considerable gender imbalance, which can be explained, on one side, by the self-selection effect, as female participants have a higher propensity to participate in psychological questionnaire surveys, and, in contrast, by the fact that normal (teacher-training) universities were among the surveyed institutions, where the baseline female student population is much larger, thereby further increasing the proportion of female respondents. The analytical dataset contained no missing data, with data available for all core variables and covariates. Statistical analyses were done as complete-case analyses without imputation of data.

### 2.3 Measures

#### 2.3.1 Mobile phone dependency index

Smartphone dependence was measured by the Chinese version of the 17-item Mobile Phone Addiction Index (MPAI). Topics include lack of control, withdrawal, avoidance, and inefficiency, with examples of difficulty controlling time on the phone, discomfort when access is blocked, using the phone to avoid problems, and phone use interfering with work that has been planned. Items are rated from 1 (not at all) to 5 (always) and total scores range from 17 to 85 with higher scores indicating more dependence-related symptoms. In this sample Cronbach’s α was 0.898.

#### 2.3.2 Short-form parenting style questionnaire

Perceived parenting styles were measured by the short-form Parenting Styles Questionnaire based on the EMBU and Chinese revised version. Participants rated fathers and mothers separately on 21 parallel items measuring rejection (6 items; e.g., perceived criticism or lack of acceptance), emotional warmth (7 items; e.g., perceived affection and encouragement), and overprotection (8 items; e.g., perceived intrusive control). The responses ranged from 1 (never) to 4 (always). Scores range from 6 to 24 for rejection, 7–28 for emotional warmth, and 8–32 for overprotection for each parent. The total for each parent ranges from 21 to 84. Participants were asked to report their perceptions of typical parent–child experiences, rather than objective observations of parenting. The fact that these retrospective perceptions were gathered simultaneously with the outcomes leaves open the possibility of recall and shared-method bias. In the current sample Cronbach’s α was 0.826.

#### 2.3.3 Self-control

Self-control was measured by the 16-item Chinese revised Self-Control Scale, which was adapted from Grasmick’s questionnaire. The scale measures impulsive risk taking, emotional control and preference for simple over effortful tasks. Representative items include acting without thinking and difficulty sticking with difficult tasks. Items are rated on a scale from 1 (not at all applicable) to 5 (fully applicable). Total scores ranged from 16 to 80 after reverse scoring of the indicated items. Higher scores indicated more self-control. Cronbach’s alpha in the present sample was 0.789.

#### 2.3.4 College students’ gratitude scale

The level of gratitude was measured by the 34-item College Students’ Gratitude Scale. Its three dimensions assess absence of deprivation, gratitude toward society, and gratitude toward nature; representative content concerns noticing benefits in daily life, appreciating support received from others, and valuing the natural environment. Items are scored on a 5-point scale, with total scores ranging from 34 to 170, where higher scores indicate higher levels of gratitude. The Cronbach’s α in this sample was 0.924.

### 2.4 Data analysis

Data was entered in EpiData and analyzed by SPSS 22.0 version. First, the data quality and potential common method bias were examined. Secondly, ranges of observed scores and descriptive statistics were calculated. Using the traditional extreme-groups approach, the high-score and low-score groups were defined only for descriptive contrast as participants in the top and bottom 27% of MPAI scores; these labels were not intended to represent clinical categories or diagnostic cutoffs. Third, Pearson correlations were calculated and paternal and maternal parenting dimensions were retained separately. For the mediation models, the respective paternal and maternal scores were aggregated at the family-dimension level (rejection, emotional warmth, and overprotection) to reduce complexity of the models. Fourth, PROCESS Model 6 was used to estimate direct, specific indirect, and serial indirect effects, with 5,000 bootstrap samples. Covariates were gender, age, hukou, and only-child status. The indirect effect was considered statistically distinguishable from zero if the 95% bootstrap confidence interval did not contain zero.

### 2.5 Ethics statement

Prior to analysis all personally identifiable information was made irreversibly anonymous and each participant was assigned a random code. The approving body was the Ethics Committee of Xi’an FanYi University (approval no. [2025]003) as mentioned in the manuscript and submission record. The submission record indicates that the participants provided written informed consent. The study procedures were planned to comply with the Declaration of Helsinki and applicable institutional requirements.

## 3 Results

### 3.1 Common method biases

The data may suffer from common method bias because all study variables were assessed using self-report questionnaires. We employed a series of procedural and statistical steps to minimise and assess this potential source of bias. Firstly, well-known scales with acceptable reliability were applied. Second, the survey was conducted anonymously to reduce evaluation apprehension and socially desirable responding. Third, Harman’s one-factor test was conducted after data collection. Nineteen factors had eigenvalues greater than 1. The first unrotated factor accounted for 19.35% of the variance, which is less than the 40% criterion used in this study. These results suggested that no one factor accounted for a large amount of the covariance among the measures.

### 3.2 Descriptive statistics and analysis of differences

The mean MPAI score was 46.51 (SD = 12.92) on a possible range of 17–85, corresponding to 52.3% of the distance from the scale minimum to maximum. This value describes the sample’s average symptom level and should not be interpreted as a clinical category because the MPAI has no diagnostic cut-off applied in this study. The observed subscale means were uncontrollability, 17.34 (SD = 5.41); withdrawal, 12.07 (SD = 2.88); avoidance, 8.40 (SD = 3.48); and inefficiency, 5.80 (SD = 2.26). To provide a descriptive contrast, participants in the upper and lower 27% of MPAI scores were labeled high-score and low-score groups. The 27% rule is a statistical extreme-groups convention that increases separation between comparison groups; it is not a validated dependence threshold. The first five rows of Table 1 describe the total sample’s MPAI total and subscale scores. The remaining rows compare parenting-style and self-control scores between the low- and high-MPAI groups to illustrate variables that differentiate the extremes of the smartphone-dependence distribution.

**Table 1:** Means, standard deviations, and *t*/*F*-test results of smartphone dependence.

Variable	Total sample M(SD)	Low-dependency group	High-dependency group	*F*/*t*
MPAI total score	46.51 ± 12.92			8.30^***^
Uncontrollability	17.34 ± 5.41			7.03^***^
Withdrawal	12.07 ± 2.88			0.41
Evasive	8.4 ± 3.48			5.31^**^
Inefficiency	5.8 ± 2.26			5.95^**^
Total parenting style score		87.84 ± 10.71	94.96 ± 12.91	−6.510^***^
Total score for father’s parenting style		43.35 ± 5.35	46.76 ± 7.29	−5.673^***^
Father rejection		7.63 ± 2.30	9.63 ± 3.799	−6.780^***^
Father’s emotional warmth		20.99 ± 4.86	19.58 ± 4.87	3.069^**^
Father’s overprotection		14.73 ± 3.58	17.54 ± 4.30	−7.556^***^
Total score for mother’s parenting style		44.49 ± 5.39	48.20 ± 6.62	−6.54^***^
Mother’s rejection		7.71 ± 2.32	9.79 ± 3.32	−7.700^***^
Mother’s emotional warmth		21.54 ± 4.59	20.20 ± 4.38	3.170^**^
Mother’s overprotection		15.24 ± 3.74	18.22 ± 4.56	−7.693^***^
Total self-control score		37.11 ± 7.35	44.10 ± 7.57	−9.947^***^
Impulsive adventurousness		7.29 ± 2.72	10.02 ± 3.24	−9.566^***^
Self-emotional		14.42 ± 3.00	16.28 ± 3.22	−6.314^***^
Simplistic tendency		15.39 ± 3.55	17.80 ± 3.18	−7.572^***^

For interpretation of the other constructs, the possible ranges were 21–84 for each parent’s total parenting-style score, 6–24 for rejection, 7–28 for emotional warmth, 8–32 for overprotection, 16–80 for self-control, and 34–170 for gratitude. Because validated categorical cut-offs are unavailable for these measures in the present sample, means are interpreted comparatively rather than as low, moderate, or high clinical classifications.

Results of difference tests indicated that smartphone dependence showed no statistically significant differences in terms of gender, family location, parental education level, or whether the participant was an only child. Detailed information is presented in Table 1.

### 3.3 Bivariate correlation analyses

The analytic level differs across tables by design. Table 1 presents descriptive extreme-group comparisons and Table 2 retains separate paternal and maternal dimensions to show parent-specific bivariate associations. Tables 3–5 report the mediation models using family-level rejection, emotional warmth, and overprotection composites formed from corresponding paternal and maternal dimension scores. This aggregation reduces model complexity and aligns the mediation analyses with the study’s family-level hypotheses; it should not be interpreted as evidence that paternal and maternal effects are identical.

**Table 2:** Descriptive statistics and matrix of correlation coefficients for each variable.

Parenting dimension	*M* ± SD	7	8	9
1. Father rejection	8.40 ± 3.00	−0.27∗∗	−0.51∗∗	0.29∗∗
2. Father’s Emotional Warmth	20.26 ± 4.66	0.28∗∗	0.52∗∗	−0.13∗∗
3. Father’s overprotection	15.95 ± 4.33	−0.29∗∗	−0.27∗∗	0.30∗∗
4. Mother’s rejection	8.57 ± 2.84	−0.27∗∗	−0.46∗∗	0.30∗∗
5. Mother’s emotional warmth	20.90 ± 4.33	0.26∗∗	0.56∗∗	−0.14∗∗
6. Mother’s overprotection	16.58 ± 4.16	0.47∗∗	−0.24∗∗	0.30∗∗
7. Self-control score	39.90 ± 8.31		0.53∗∗	−0.47∗∗
8. Gratefulness	5.76 ± 0.78			−0.49∗∗
9. Mobile Phone Dependency	45.02 ± 13.41			

^*^ denotes *p* < 0.05, ^**^ denotes *p* < 0.01, and ^***^ denotes *p* < 0.001. The same notation is used below.

**Table 3:** Chain-mediated effects of self-control, gratitude between parental rejection and cell phone dependence.

Trails	Agency effect	Effect size	Standard error	CI
Parental rejection—self-control-mobile phone dependence	0.122	28.5%	0.04	0.059–0.120
Parental rejection—self-control-gratitude-mobile phone dependency	0.010	2.4%	0.01	0.002–0.026
Parental rejection—gratitude-mobile phone dependency	0.027	6.2%	0.02	0.005–0.069

**Table 4:** Chain-mediated effects of self-control, gratitude between parental overprotection and cell phone dependence.

Trails	Agency effect	Effect size	Standard error	CI
Parental overprotection—self-control-mobile phone dependence	0.194	37.0%	0.04	0.123–0.279
Parental overprotection—self-control-gratitude-mobile phone dependency	0.012	2.3%	0.01	0.001–0.030
Parental overprotection—gratitude-mobile phone dependency	0.044	8.5%	0.03	0.005–0.111

**Table 5:** Chain-mediated effects of self-control, gratitude between parental emotional warmth and cell phone dependence.

trails	Agency effect	Effect size	Standard error	CI
Parental emotional warmth—self-control-mobile phone dependence	0.122	28.5%	0.04	0.059–0.120
Parental emotional warmth—self-control-gratitude-mobile phone dependency	0.010	2.4%	0.01	0.002–0.026
Parental emotional warmth—gratitude-mobile phone dependency	0.027	6.2%	0.02	0.005–0.069

Table 2 presents the means and standard deviations (*M* ± SD) along with Pearson correlation coefficients among nine variables: paternal rejection, paternal emotional warmth, paternal overprotection, maternal rejection, maternal emotional warmth, maternal overprotection, self-control, gratitude, and smartphone dependence. The results showed that paternal rejection was significantly negatively correlated with self-control (*r* = −0.27, *p* < 0.01) and gratitude (*r* = −0.51, *p* < 0.001), and significantly positively correlated with smartphone dependence (*r* = 0.29, *p* < 0.001); paternal emotional warmth was significantly positively correlated with self-control (*r* = 0.28, *p* < 0.01) and gratitude (*r* = 0.52, *p* < 0.001), and significantly negatively correlated with smartphone dependence (*r* = −0.13, *p* < 0.001); paternal overprotection was significantly negatively correlated with self-control (*r* = −0.29, *p* < 0.01) and gratitude (*r* = −0.27, *p* < 0.001), and significantly positively correlated with smartphone dependence (*r* = 0.30, *p* < 0.001); maternal rejection was significantly negatively correlated with self-control (*r* = −0.27, *p* < 0.01) and gratitude (*r* = −0.46, *p* < 0.001), and significantly positively correlated with smartphone dependence (*r* = 0.30, *p* < 0.001); maternal emotional warmth was significantly positively correlated with self-control (*r* = 0.26, *p* < 0.01) and gratitude (*r* = 0.56, *p* < 0.001), and significantly negatively correlated with smartphone dependence (*r* = −0.14, *p* < 0.001); maternal overprotection was significantly positively correlated with self-control (*r* = 0.47, *p* < 0.001), significantly negatively correlated with gratitude (*r* = −0.24, *p* < 0.001), and significantly positively correlated with smartphone dependence (*r* = 0.30, *p* < 0.001); self-control was significantly positively correlated with gratitude (*r* = 0.53, *p* < 0.001) and significantly negatively correlated with smartphone dependence (*r* = −0.47, *p* < 0.001); gratitude was significantly negatively correlated with smartphone dependence (*r* = −0.49, *p* < 0.001).

### 3.4 Analysis of the serial mediation effects

PROCESS Model 6 (Hayes, 2013) was used to test serial mediation. PROCESS provides estimates of direct and indirect effects in models with multiple mediators and covariates, and bootstrap confidence intervals for inference. In this work 5,000 bootstrap samples were used. Covariates were gender, age, place of origin and only child status. Serial indirect effects of self-control and gratitude were tested separately for each dimension of parenting.

#### 3.4.1 Serial mediating effects of self-control and gratitude between parental rejection and smartphone dependence

The results in Table 3 and Figure 2 show that the direct effect of parental rejection on college students’ mobile phone dependence is 0.268 (*p* = 0.002), and the 95% confidence interval does not include 0 (LLCI = 0.099, ULCI = 0.437). The mediating paths of parental rejection: self-control, mobile phone dependence, parental rejection, gratitude-mobile phone dependence, and parental rejection, gratitude, mobile phone dependence are all significant (the confidence intervals do not include 0). The total mediating effect is 0.122 + 0.010 + 0.027 = 0.159; the total effect is 0.268 + 0.159 = 0.427; and the effect size of the total mediating effect is 37.1%. The results indicate that there is one direct effect path and three mediating effect paths between parental overprotection and college students’ mobile phone dependence.

**Figure 2:**
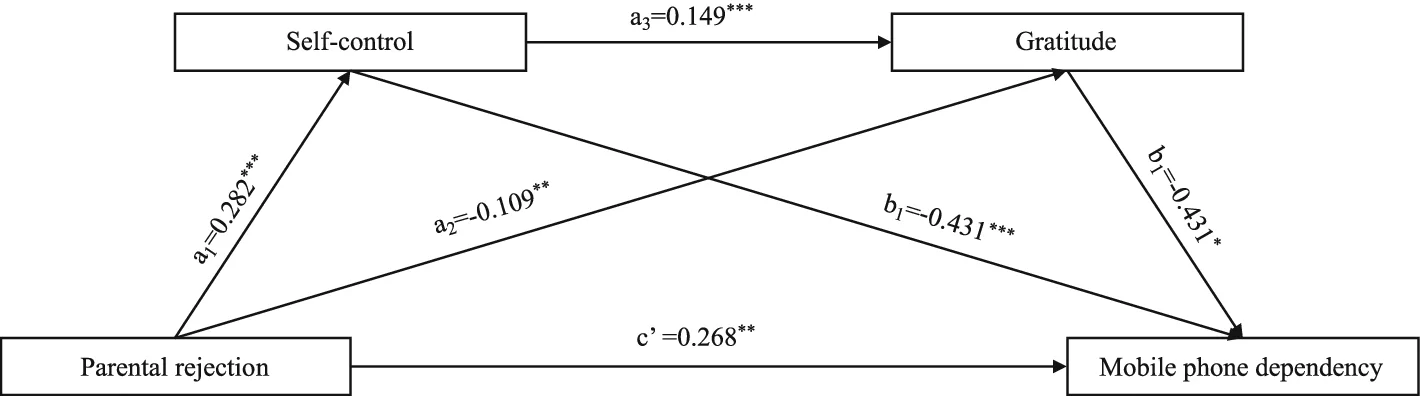
Chain-mediated effects of self-control, gratitude between parental rejection and mobile phone dependence.

#### 3.4.2 Serial mediating effects of self-control and gratitude between parental overprotection and smartphone dependence

The results in Table 4 and Figure 3 show that the direct effect of parental overprotection on college students’ mobile phone dependence is 0.274 (*p* = 0.004), and the 95% confidence interval does not include 0 (LLCI = 0.087, ULCI = 0.462). The three mediating paths of parental overprotection: (i) self-control: mobile phone dependence, parental overprotection; (ii) self-control: gratitude—mobile phone dependence, and (iii) parental overprotection: gratitude; mobile phone dependence are all significant (the confidence intervals do not include 0). The total mediating effect is 0.194 + 0.012 + 0.044 = 0.250; the total effect is 0.274 + 0.250 = 0.524; and the effect size of the total mediating effect is 47.7%. The results indicate that there is one direct effect path and three mediating effect paths between parental rejection and college students’ mobile phone dependence.

**Figure 3:**
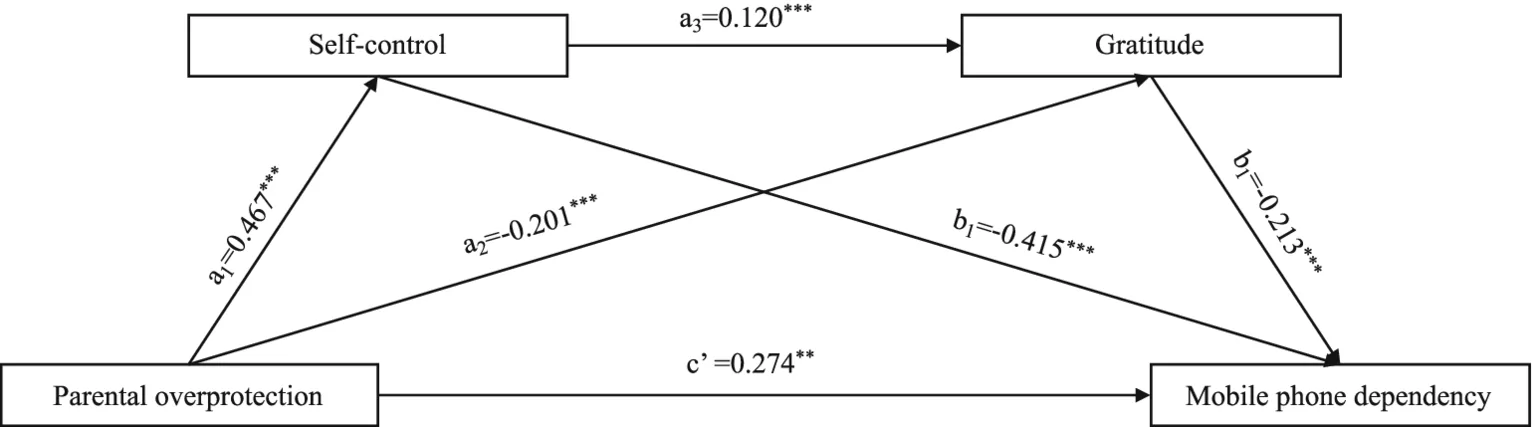
Chain-mediated effects of self-control, gratitude between parental overprotection and mobile phone dependence.

#### 3.4.3 Serial mediating effects of self-control and gratitude between parental emotional warmth and smartphone dependence

The results in Table 5 and Figure 4 show that the direct effect of parental emotional warmth on college students’ mobile phone dependence is 0.015 (*p* = 0.820), and the 95% confidence interval contains 0 (LLCI = −0.118, ULCI = 0.149). The total mediating effect is −0.111 + (−0.010) + (−0.034) = −0.155; the total effect is 0.015 + (−0.155) = −0.140; and the effect size of the total mediating effect is 37.1%. The results indicate that there are only three mediating effect paths between parental emotional warmth and college students’ mobile phone dependence.

**Figure 4:**
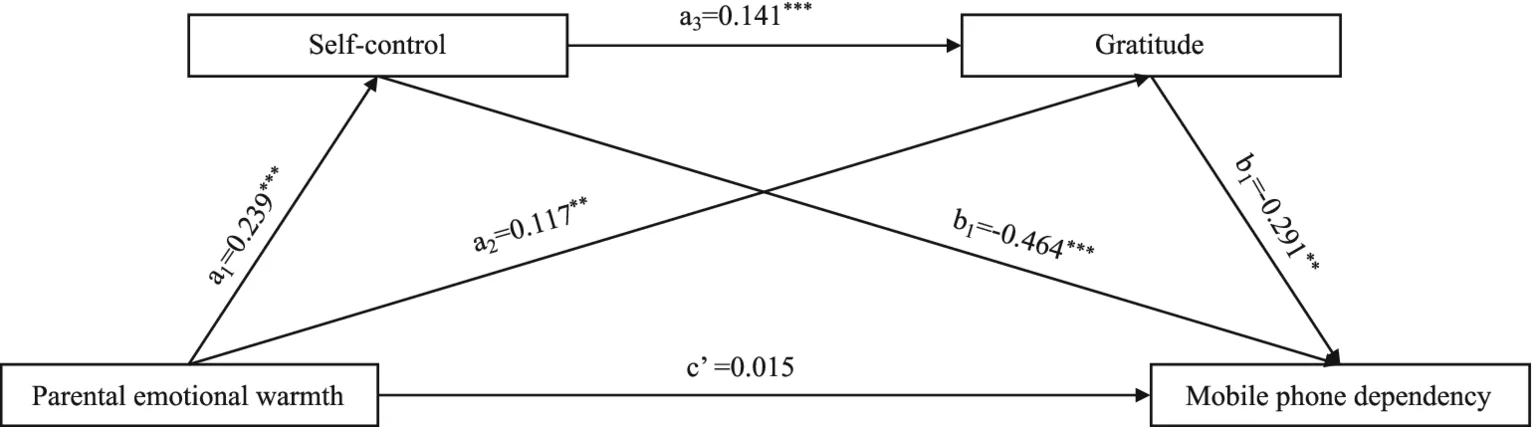
Chain-mediated effects of self-control, gratitude between parental emotional warmth and mobile phone dependence.

## 4 Discussion

Parental rejection and overprotection had significant positive direct effects on smartphone dependence (direct effects = 0.268 and 0.274, respectively, *p* < 0.01). However, the direct effect of emotional warmth was not statistically significant (effect = 0.015, *p* > 0.05). These results are generally consistent with Problem Behavior Theory that connects perceived environmental conditions, personality characteristics and behavioral outcomes (Zhen et al., 2019). Negative family experiences may be associated with lower psychological security and poor self-regulation, which may result in increased reliance on smartphones as an accessible coping resource. For instance, students who experience higher parental rejection may be more likely to seek reassurance or distraction thru smartphone-mediated activities when their emotional needs are not met (Eyuboglu et al., 2021). Similarly, overprotective parenting may restrict opportunities for autonomous decision-making and coping, potentially increasing reliance on smartphone entertainment during academic or interpersonal stress (Zhen et al., 2019). These mechanisms remain interpretive because the data presented are cross-sectional.

These findings are consistent with studies linking negative parenting, insecure parental bonding, and controlling family climates to problematic smartphone use (He et al., 2024; Lian et al., 2016; Xin et al., 2022). However, alternative explanations remain plausible. Students who experience current distress or problematic smartphone use may evaluate past parenting more negatively, and unmeasured factors such as peer conflict, academic workload, personality, or family socioeconomic conditions may influence both perceived parenting and dependence. The cross-sectional associations should therefore be interpreted as compatible with, rather than proof of, the proposed developmental direction.

It is noteworthy that the direct effect of parental emotional warmth on smartphone dependence is not significant. This result does not negate its influence but rather reveals the “indirect effect preference” of positive parenting styles emotional warmth indirectly exerts a protective effect by shaping positive psychological traits. This is consistent with the perspective of the “Ecological Systems Theory”, which emphasizes that the family microsystem influences individual development through internalization mechanisms. Positive parenting does not directly intervene in behavior but constructs a secure psychological environment that fosters stable self-regulation abilities and positive cognitive patterns, enabling students to autonomously resist the temptation of smartphone dependence (Guan et al., 2025). This “indirect effect dominance” suggests that the inhibitory effect of positive parenting on smartphone dependence is “long-lasting” and “fundamental”, permeating deep dimensions of psychological functioning rather than remaining superficial.

From the correlation results, parental rejection and overprotection showed correlation coefficients of 0.29 and 0.30 (*p* < 0.001) with smartphone dependence, while emotional warmth showed a correlation coefficient of −0.13 (*p* < 0.001). This further validates the pattern that “the type of parenting style determines the direction of influence.” This finding addresses the limitation of existing research, which often emphasizes the direct effects of negative parenting while overlooking the indirect pathways of positive parenting. It clarifies a “dual-path model” of parenting styles’ influence on smartphone dependence: negative parenting operates through both “direct and indirect” pathways, whereas positive parenting functions solely through an “indirect” pathway (Nwefuru et al., 2019). This provides empirical evidence for distinguishing the operational modes of parenting styles in future research.

This study confirms that self-control plays a significant mediating role between parenting styles and smartphone dependence among college students: parental rejection (*β* = −0.27, *p* < 0.001) and overprotection (*β* = −0.29, *p* < 0.001) negatively predict self-control, while emotional warmth (*β* = 0.28, *p* < 0.001) positively predicts self-control. Furthermore, self-control negatively predicts smartphone dependence (*β* = −0.47, *p* < 0.001). This chain of transmission fully aligns with the “observational learning-behavior internalization” mechanism of Social Learning Theory (Rose, 2024), which posits that an individual’s self-control ability is not innate but gradually internalized through observing parental behaviors and imitating regulatory strategies.

The self-control result converges with evidence that weak inhibitory control and depleted regulatory resources increase vulnerability to problematic smartphone use (Servidio, 2019; Zhong et al., 2025). It also extends the parenting–self-control literature by placing self-control between perceived parenting and smartphone dependence (Finkenauer et al., 2005; Li et al., 2019). Nevertheless, self-control may also be eroded by repeated smartphone habits, sleep disruption, or current stress, suggesting possible reciprocal effects that require longitudinal testing.

Specifically, parental emotional warmth fosters self-control in college students through “supportive exploration.” In an emotionally warm family environment, parents provide moderate guidance while allowing students to autonomously explore. This “autonomy + support” parenting model enables students to learn delay of gratification and impulse inhibition through practice, gradually forming stable self-control traits. In contrast, parental rejection and overprotection undermine self-control development through “negative feedback” and “controlling interventions” (Tangney et al., 2004). Frequent parental negation of students’ behaviors weakens their self-efficacy, diminishing their confidence in behavioral regulation. Overprotection directly deprives them of opportunities to practice self-regulation, making it difficult for students to mobilize psychological resources to resist immediate gratification when faced with smartphone temptations (Wang and Qi, 2017). This ultimately traps them in a cycle of “impulsive use that progresses to loss of control and dependence” (Khang et al., 2013).

In terms of mediating effect sizes, self-control accounted for 28.5, 37.0, and 28.5% of the mediating effects between parental rejection, overprotection, emotional warmth, and smartphone dependence, respectively, representing the largest effects among all mediating pathways. This indicates that self-control is the “core mediating variable” through which parenting styles influence smartphone dependence. This finding supports the perspective of the “Self-Control Resource Model” (Akn et al., 2015; Baumeister et al., 2006), individual behavior relies on self-control resources, and family upbringing serves as a critical “supply source” of these resources. Negative parenting depletes self-control resources, while positive parenting replenishes them. The abundance or scarcity of these resources directly determines an individual’s ability to resist smartphone dependence (Finkenauer et al., 2005).

This study reveals that gratitude plays a significant mediating role between parenting styles and smartphone dependence among college students: parental rejection (*β* = −0.51, *p* < 0.001) and overprotection (*β* = −0.27, *p* < 0.001) negatively predict gratitude, while emotional warmth (*β* = 0.52, *p* < 0.001) positively predicts gratitude. Furthermore, gratitude negatively predicts smartphone dependence (*β* = −0.49, *p* < 0.001). This mechanism can be well explained by the “Broaden-and-Build Theory of Positive Emotions” (Fredrickson, 2001a, b), which posits that gratitude, as a typical positive emotion, broadens individuals’ cognitive scope and behavioral repertoire, facilitating the construction of enduring psychological resources, thereby reducing the need for negative behaviors such as smartphone dependence.

The gratitude pathway accords with research showing that gratitude is associated with lower addictive tendencies and may redirect attention toward valued offline relationships and long-term goals (Gao et al., 2023; Zhang et al., 2024). At the same time, gratitude may partly reflect broader wellbeing, social support, or positive affect. These overlapping resources were not modeled simultaneously, so the observed indirect effect should not be treated as unique to gratitude without further comparative research.

Regarding the transmission pathway from parenting styles to gratitude, parental emotional warmth shapes college students’ gratitude traits through “emotional transmission-cognitive internalization.” Consistent parental care and support enable students to persistently perceive themselves as “loved” and “treated with kindness.” These positive experiences gradually internalize into a positive cognitive bias toward the world; a belief that “there is much beauty and kindness in real life,” thereby forming a gratitude trait characterized by actively noticing, acknowledging, and reciprocating kindness (Barnett et al., 2021; Peng et al., 2025). Neuroscientific research provides physiological evidence for this mechanism: Garcia-Vazquez et al. (2020) used brain imaging to demonstrate that maternal emotional warmth is positively correlated with the volume of the rostro lateral prefrontal cortex in college students, indicating that positive parenting may promote the formation of gratitude traits through neural development.

Regarding the inhibitory pathway from gratitude to smartphone dependence, students with high levels of gratitude resist smartphone dependence through a triple mechanism (Ashwathi and Hrithika, 2024; Barnett et al., 2021; Zhang et al., 2024): First, gratitude broadens cognitive scope, enabling students to focus more on positive events in real life, reducing their reliance on virtual rewards from smartphones. Second, gratitude promotes real-world interpersonal connections. Highly grateful individuals are more inclined to actively maintain offline relationships, and high-quality real-world social interactions diminish their motivation to seek belonging through virtual social interactions on smartphones. Third, gratitude alleviates negative emotions. By activating positive emotions, gratitude counteracts negative emotions such as anxiety and boredom that trigger smartphone dependence, reducing the behavioral impulse to “escape emotions through smartphones” (Gao et al., 2023). The strong negative correlation between gratitude and smartphone dependence (*r* = −0.49, *p* < 0.001) observed in this study is a result of the combined action of these three mechanisms.

The core finding of this study is the validation of the serial mediating effect of “parenting styles influence self-control, which shapes gratitude and subsequently affects smartphone dependence,” which was significant across all three parenting styles (effect sizes: 2.4% under rejection, 2.3% under overprotection, and 2.4% under emotional warmth). This result reveals the “progressive transmission logic” through which parenting styles influence college students’ smartphone dependence self-control, as a “foundational regulatory ability,” provides the prerequisite for the formation of gratitude, an “positive personality trait.” Together, they form a dual protective barrier of “ability-trait” or a dual risk chain of “deficiency-weakness.” This mechanism can be explained through an integrated framework of the “Resource Accumulation Theory” and the “Dual-System Model of Self-Regulation” (Hobfoll, 2002).

The relatively small serial indirect effects indicate that this pathway is statistically detectable but represents only one component of a multifactorial process. The model complements rather than replaces explanations based on current peer support, negative emotion, fear of missing out, platform design, and habitual reinforcement (Guo, J., et al., 2025; Song et al., 2025; Zhong et al., 2025). Its contribution lies in showing how a distal perceived family context and two modifiable psychological resources can be examined within one coherent pathway.

The serial pathway of emotional warmth is consistent with a positive sequence in which supportive parenting is linked to stronger self-control, stronger self-control is linked to greater gratitude, and greater gratitude is linked to lower smartphone dependence. Greater self-control may enable individuals to suppress immediate impulses and focus on long-term interpersonal benefits, which may promote grateful responses (DeSteno et al., 2014). Gratitude, in turn, may facilitate engagement with meaningful offline activities and relationships (Ashwathi and Hrithika, 2024). These interpretations are consistent with the observed associations, but need to be confirmed with longitudinal or experimental studies.

In line with this, the serial pathway of rejection and overprotection was in the opposite direction: less supportive family experiences were related to lower self-control, which was related to lower gratitude and then higher smartphone dependence. Self-regulatory difficulties could focus attention on immediate needs and rewards, and lower gratitude may go with less active involvement in valued offline relationships (Ent et al., 2015; Barnett et al., 2021). Because the present study did not establish temporal precedence, these proposed processes should be viewed as explanatory hypotheses rather than as demonstrated causal sequences.

The serial mediation results add to past work that has often examined self-control and gratitude as separate mediators. Based on the present model, the two variables can also be examined in one sequential pathway that connects perceived parenting experiences to smartphone dependence (Ashwathi and Hrithika, 2024; Garcia-Vazquez et al., 2020). However, the relatively small serial indirect effects suggest this pathway should be interpreted as one component of a broader, multifactorial process rather than a complete explanation. The larger total effect for overprotection (0.524) than for rejection (0.427) should also be interpreted as a sample-specific finding, rather than as evidence of a universal ordering of parenting effects.

## 5 Research limitations and future prospects

There are several limitations in this study. First, the cross-sectional design does not establish the temporal precedence of the variables and their causal relationships. Longitudinal studies are needed to confirm the proposed sequence of parenting experiences, self-control, gratitude, and smartphone dependence over time. Second, the study used non-probability convenience sampling, and the sample was predominantly female (68.0%) which limits representativeness and external generalisability. We adjusted for differences in observable characteristics by including demographic covariates, but statistical adjustment cannot address the self-selection bias arising from non-random recruitment. Future research should utilise probability-based sampling whenever possible, or utilise stratified recruitment to achieve a more balanced and diverse sample. Third, this study did not distinguish between different types of smartphone use, such as learning use, social use, gaming use, and entertainment use. Future research should investigate whether the proposed associations differ across particular usage patterns and contexts.

## 6 Conclusion

Perceived parental rejection and overprotection were positively associated with smartphone dependence, whereas emotional warmth operated primarily through indirect pathways. Self-control and gratitude each mediated the parenting-dependence relationship, and their statistically significant serial pathway linked perceived family experiences with a proximal digital behavior. The findings support an integrated view in which family context, self-regulatory capacity, positive cognitive-affective resources, and current social conditions jointly contribute to problematic smartphone use. Interventions should therefore avoid treating dependence as a matter of willpower alone and instead combine autonomy-supportive family communication, self-regulation training, gratitude or relationship-strengthening activities, and support for current academic and interpersonal stress. Because the data are cross-sectional and nonrepresentative, these implications should be tested in longitudinal and more diverse samples.

## Data Availability

The raw data supporting the conclusions of this article will be made available by the authors, without undue reservation.
